# The CD14 C-260T single nucleotide polymorphism (SNP) modulates monocyte/macrophage activation in treated HIV-infected individuals

**DOI:** 10.1186/s12967-015-0391-6

**Published:** 2015-01-27

**Authors:** Reena Rajasuriar, Yong Yean Kong, Reshika Nadarajah, Noor Kamila Abdullah, Tim Spelman, Muhamad Yazli Yuhana, Sasheela Ponampalavanar, Adeeba Kamarulzaman, Sharon R Lewin

**Affiliations:** Department of Pharmacy, Faculty of Medicine, University of Malaya, 50603 Kuala Lumpur, Malaysia; Centre of Excellence for Research in AIDS (CERiA), University of Malaya, 50603 Kuala Lumpur, Malaysia; Department of Infectious Diseases, Monash University and Alfred Hospital, 3004 Melbourne, Australia; Centre for Population Health, Burnet Institute, 3004 Melbourne, Australia; Department of Medicine, Faculty of Medicine, University of Malaya, 50603 Kuala Lumpur, Malaysia; Faculty of Medicine, University Teknologi MARA, 47000 Sungai Buloh, Selangor Malaysia; Centre for Biomedical Research, Burnet Institute, 3004 Melbourne, Australia; Doherty Institute for Infection and Immunity, The University of Melbourne, Melbourne, 3010 Australia

**Keywords:** HIV, Lipopolysaccharide, CD12 C-260T, Soluble CD14, Soluble CD163, Monocyte activation, C-reactive protein, Atherosclerosis, Carotid intima media thickness

## Abstract

**Background:**

HIV-infected individuals have an increased risk of cardiovascular disease (CVD). T-allele carriers of the CD14 C-260T single-nucleotide polymorphism (SNP) have reported increased expression of the LPS-binding receptor, CD14 and inflammation in the general population. Our aim was to explore the relationship of this SNP with monocyte/macrophage activation and inflammation and its association with sub-clinical atherosclerosis in HIV-infected individuals.

**Methods:**

Patients with no pre-existing CVD risk factors on suppressive antiretroviral therapy were recruited from University Malaya Medical Centre, Malaysia (n = 84). The CD14 C-260T and TLR4 SNPs, Asp299Gly and Thr399Ile were genotyped and soluble(s) CD14 and sCD163 and high-sensitivity C-reactive protein, hsCRP were measured in plasma. Subclinical atherosclerosis was assessed by measuring carotid intima media thickness (cIMT). The association between CD14 C-260T SNP carriage and cIMT was assessed in a multivariable quantile regression model where a p-value of <0.05 was considered significant.

**Results:**

We found the CD14 C-260T T-allele in 56% of the cohort and evidence of subclinical atherosclerosis in 27%. TT genotype was associated with higher sCD163 (p = 0.009) but only marginally higher sCD14 (p = 0.209) and no difference in hsCRP (p = 0.296) compared to CC/CT. In multivariable analysis, only Framingham risk score was independently associated with higher cIMT while lower sCD163 was trending towards significance. No association was found in TT-genotype carriers and cIMT measurements.

**Conclusion:**

The CD14 C-260T SNP was associated with increased monocyte activation but not systemic inflammation or cIMT in this HIV-infected cohort with low CVD risk profile.

## Background

Persistent immune activation and inflammation have been well described in chronic HIV disease (reviewed in [1]) and have been associated with an increased risk of atherosclerosis in this population [2-6]. Many factors contribute to chronic immune activation including the translocation of microbial products including lipopolysaccharide (LPS) from damaged gut-associated lymphoid tissue sustained during early HIV disease [7,8]. Chronic elevation of LPS persists despite suppressive combination antiretroviral therapy (cART) [7,9] and may drive increased cardiovascular disease (CVD) risk in HIV-infected individuals [2,10] as has been found in HIV-negative populations [11-13].

CD14 is a co-receptor for LPS, and together with MD-2 binds to toll-like receptor 4 (TLR4) on monocytes and macrophages activating them (reviewed in [14]). There is also a soluble form for CD14 (sCD14) which is secreted by monocytes/macrophages following LPS stimulation and by liver cells as an acute-phase protein following stimulation by IL-6 [15]. Soluble CD14 plays an important role in LPS-mediated activation and injury of cells lacking membrane-bound CD14 including endothelial and epithelial cells [16].

A common polymorphism in the CD14 gene, C-260T has been associated with differential expression levels of CD14 on monocytes/macrophages [17-19]. The SNP (C → T) occurs in the promoter region of the CD14 gene and increases the binding affinity of specificity protein (Sp) transcription factors [20], leading to subsequent increases in CD14 production [20,21]. T vs C allele carriers have been found to have higher density of membrane-bound CD14 and circulating levels of sCD14 [18,19,22] and subsequent increased production of pro-inflammatory cytokines following LPS stimulation [19,21,23]. This in turn may influence the development and progression of atherosclerotic disease. Conversely, two non-synonymous SNPs in the TLR4 gene, Asp299Gly and Thr399Ile, have been associated with reduced cytokine responses following LPS stimulation in some studies [24,25] and the carriage of these SNPs may also modulate LPS-mediated responses.

In genotype-phenotype studies, the association between the C-260T single nucleotide polymorphism (SNP) and cardiovascular disease have been mixed with some studies reporting a positive association while others have not [18,22,26-31]. Two comprehensive meta-analyses have however suggested that this association may be more relevant in East Asians compared to Caucasians [32,33].

Given the association between LPS-mediated monocyte/macrophage activation and CVD in HIV-infected individuals [10,34,35], we hypothesised that the CD14 (C-260T) SNP would be associated with increased risk of CVD in an Asian cohort of HIV-infected individuals. To help better delineate the potential influence of this SNP from other metabolic processes known to increase CVD risk, the study was conducted in patients who reported no clinical history of CVD risk factors (IHD, diabetes, hypertension or dyslipidemia) at recruitment. Our aims were 1) to explore the relationship between the CD14 C-260T SNP on markers of monocyte/macrophage activation (including soluble(s) CD14 and sCD163) and inflammation (as measured by highly sensitive C-reactive protein (hsCRP) and 2) to assess the association between the CD14 C-260T SNP and subclinical atherosclerosis measured by carotid intima-media thickness (cIMT).

## Methods

### Study population

This was a sub-study of a clinic-based cohort that was established to study the prevalence of metabolic syndrome and subclinical atherosclerosis among HIV-infected individuals attending the University Malaya Medical Centre (UMMC), Malaysia. Participants in the sub-study were re-consented for genetic testing. The inclusion criteria were; receiving cART for a minimum duration of 6 months, no evidence of symptomatic AIDS in the last 6 months, no pre-existing clinical history of CVD or other atherosclerotic disease including cerebrovascular or peripheral vascular disease, pregnancy, hypertension, diabetes mellitus, dyslipidemia and malignancies.

Subclinical atherosclerosis was assessed by measurement of cIMT with high-resolution ultrasonography (Philips IU-22, USA) at 6 sites; right and left mid common carotid arteries, carotid bifurcation and the proximal site of the internal carotid arteries. Measurements of cIMT from left and right arteries were pooled and mean values were used for each patient. Subclinical atherosclerosis was defined as a mean cIMT > 0.7 mm as previously described [36], the presence of plaque (focal echogenic structure with cIMT > 1.2 mm) or both. Additionally, screening for traditional cardiovascular risk factors including fasting blood glucose, lipid profile, anthropometric measurements, resting blood pressure and assessment of smoking status and family history for premature cardiovascular disease (CVD) were performed. Data on HIV-specific characteristics including HIV RNA, CD4 T-cell counts, antiretroviral drug history and history of co-infections were obtained from patient medical records.

The study was approved by the hospitals institutional review board (MEC 975.6).

### Specimen collection, genotyping and measurement of immune activation markers

Whole blood was collected in EDTA-vacutainers. Peripheral blood mononuclear cells (PBMCs), plasma and neutrophils were isolated as previously described [37]. DNA was extracted using the Qiagen Blood and Tissue kit (Qiagen, Germany) from neutrophils and genotyped for the CD14 (rs2569190) and TLR4 (rs4986790 and rs2569190) SNPs at the Australian Genome Research Facility (AGRF) using the Sequenom MassArray platform. Markers of monocyte activation including sCD14 (R&D Systems, McKinley Place, MN) and sCD163 (Trilium Diagnostics, Bangor, ME) were measured by ELISA in plasma according to the manufacturer’s instructions. The systemic inflammation marker, hsCRP was determined by immunochemiluminometric assay by the hospitals clinical diagnostic laboratory.

### Statistical analysis

Categorical variables were summarized using frequency and percentage. Continuous variables were tested for skew using a Shapiro-Wilk test and summarized using mean and standard deviation (SD) or median and inter-quartile range (IQR) as appropriate. Chi-square analysis was used to test Hardy-Weinberg equilibrium (HWE). The influence of CD14 C-260T genotypes on markers of immune activation, inflammation and cardiovascular risk factors were compared using Mann Whitney U or chi-square test. A dominant genetic model of association was chosen as the primary model of analysis given prior reports of a higher odds of SNP association when using this model compared to a recessive model in cohorts of East Asian ethnicity [33]. Median quantile regression analysis was then used to assess the independent influence of the CD14 SNP on cIMT adjusting for traditional cardiovascular risk factors and HIV-related parameters. Using quantile regression to model median cIMT was preferred over simple regression of the mean secondary to significant skew in cIMT which was not able to be corrected using standard transformations (log-, square-root or inverse Gaussian transformations). As such the key underlying assumption for outcome variable normality required by regression models of the mean was not able to be satisfied. A quantile regression by comparison does not assume underlying normality and is thus far more robust to non-normal errors and outliers. Parameters were log transformed if they were significantly skewed or if their effect sizes (coefficients) were too small or clinically meaningless if left untransformed. Co-variates for the multivariable model were selected based on a semi-backwards/semi-frontwards/semi-*a priori* modelling approach where the *a priori* assumption was that covariates significant at a p < 0.20 level in the univariable model was considered a candidate predictor to be included in the multivariable analysis. All statistical analysis was performed using Stata version 12 (StataCorp, College Station, Texas).

## Results

### Cohort characteristics and distribution of CD14 (C-260T) SNP

Patients were identified from a pre-existing study assessing the prevalence of metabolic syndrome and subclinical atherosclerosis among HIV-infected individuals on cART. A total of 84 patients consented for genetic testing from the initial cohort of 126. Most patients were male with a median (IQR) age of 41 (36 -46) years (Table 1). All patients had received cART for a median (IQR) duration of 4 (1-8) years. The majority received non-nucleoside reverse transcriptase inhibitor (NNRTI)-based regimens (88%) while 11% received a protease inhibitor (PI)-based regimen. Only one patient had prior exposure to the nucleoside reverse transcriptase inhibitor (NRTI), abacavir (1.1%) and three with a PI-based regimen (4%). The cohort in general had low CVD risk with the median (IQR) 10-year Framingham risk score of 5% (3–11). Twenty seven percent of the cohort had evidence of subclinical atherosclerosis defined by an increase in mean cIMT of >0.7 mm or the presence of plaque.

**Table 1:** **Cohort characteristics according to the CD14 C-260T SNP distribution**

**Patient characteristics**	**CD14 C-260T genotype** ^**#**^			Total cohort (N = 84)*
	CC (n = 15)*	CT (n = 41)*	TT (n = 24)*	
Age, years	38 (32–48)	41 (37–45)	41 (36–48)	41 (36–46)
Gender, n (%) male	13 (86.7%)	33 (80.5%)	17 (70.8%)	67 (79.8%)
**Ethnicity, n (%)**				
- Malay	7 (46.7%)	7 (17.1%)	3 (12.5%)	19 (22.6%)
- Chinese	7 (46.7%)	28 (68.3%)	19 (79.2%)	56 (66.7%)
- Indian	1 (6.7%)	6 (14.6%)	2 (8.3%)	9 (10.7%)
Baseline CD4 T-cell count, cells/μl	30 (8–253)	106 (13–223)	60 (22–197)	63 (16–228)
Current CD4 T-cell count, cells/μl	300 (155–450)	386 (286–536)	468 (292–631)	387 (271–547)
Current CD4:CD8 ratio	0.36 (0.17–0.55)	0.54 (0.33–0.73)	0.59 (0.28–0.79)	0.51 (0.28–0.74)
Baseline HIV RNA, copies/ml	163693 (59611–559681)	137600 (50158–316679)	105659 (33069–404288)	136423 (52214–398660)
Duration on ARV, years	2 (1–6)	5 (3–10)	7 (2–10)	4 (1–8)
**Current ARV regimen, n (%)**				
- NNRTI-based	13 (86.7%)	36 (87.8%)	21 (87.5%)	74 (88.1%)
- PI-based	2 (13.3%)	5 (12.2%)	3 (12.5%)	10 (11.9%)
Previous abacavir use, n (%)		1 (2.4%)		1 (1.1%)
Previous PI use, n (%)		3 (7.3%)		3 (3.6%)
Positive hepatitis C antibody, n (%)	0 (0.0%)	0 (0.0%)	1 (4.2%)	2 (2.4%)
Positive hepatitis B surface antigen, n (%)	0 (0.0%)	0 (0.0%)	0 (0.0%)	0 (0.0%)
**Smoking,** n (%)				
- Never	9 (60.0%)	24 (58.5%)	12 (50.0%)	46 (54.8%)
- Former	2 (13.3%)	4 (9.8%)	4 (16.7%)	10 (11.9%)
- Current	4 (26.7%)	13 (31.7%)	8 (33.3%)	28 (33.3%)
Framingham risk score, %	5.85 (1.88–11.45)	5.05 (2.93–8.63)	4.75 (2.23–14.80)	5.00 (2.75–11.05)
Body mass index, kg/m^2^	22.3 (19.8–25.1)	22.6 (21.4–25.4)	24.1 (20.8–26.2)	23.0 (21.2–25.5)
Family history CVD, n (%)	1 (6.7%)	7 (17.1%)	0 (0.0%)	8 (9.5%)
Diabetes, n (%)	0 (0.0%)	1 (2.4%)	2 (8.3%)	4 (4.8%)
Hypertension, n (%)	9 (60.0%)	14 (34.1%)	8 (33.3%)	32 (38.1%)
Fasting glucose, mmol/L	4.8 (4.5–5.5)	5.2 (4.9–5.5)	5.3 (4.7–5.8)	5.1 (4.7–5.6)
Total cholesterol, mmol/L	5.4 (4.2–5.8)	5.2 (4.7–6.2)	5.2 (4.8–5.6)	5.2 (4.6–5.8)
LDL, mmol/L	2.8 (2.5–3.6)	3.1 (2.5–3.9)	3.1 (2.6–3.5)	3.0 (2.5–3.6)
HDL, mmol/L	1.2 (0.9–1.5)	1.2 (1.0–1.5)	1.1 (0.9–1.3)	1.2 (0.9–1.4)
Triglycerides, mmol/L	2.0 (1.4–2.8)	1.8 (1.5–2.3)	2.1 (1.5–3.3)	1.9 (1.4–2.5)
Increased cIMT, (>0.7 mm), n (%)	3 (20.0%)	13 (31.7%)	6 (25.0%)	23 (27.4%)
**TLR4 frequency,** n (%)				
- TLR4 Asp299Gly (c.896A > G) (rs4986790)		1 (2.4%)		1 (1.1%)
- TLR4 Thr399Ile (c.1196C > T) (rs2569190)		1 (2.4%)		1 (1.1%)

*Data shown are median (IQR) unless otherwise stated; ^#^4 patients did not have CD14 C-260T genotype data; ARV: antiretroviral therapy, NNRTI: non-nucleoside reverse transcriptase inhibitors, PI: protease inhibitors; CVD: cardiovascular disease; LDL: low-density lipoprotein; HDL: high-density lipoprotein, cIMT: carotid intima media thickness, TLR4: toll-like receptor 4.

T-allele carriage for the CD14 (C-260T) SNP was 56% and the genotype distribution was in HWE (p = 0.718). The genotype call rate was 95.2%. We additionally genotyped the TLR4 Asp299Gly and Thr399Ile SNPs to address possible gene-gene interactions with the CD14 (C-260T) SNP but found only one patient heterozygous for the TLR4 SNPs while the remaining were wild type carriers. The TLR4 SNPs were therefore not considered in subsequent analysis.

### Association between CD14 C-260T SNP and markers of monocyte activation and systemic inflammation

**Figure 1:**
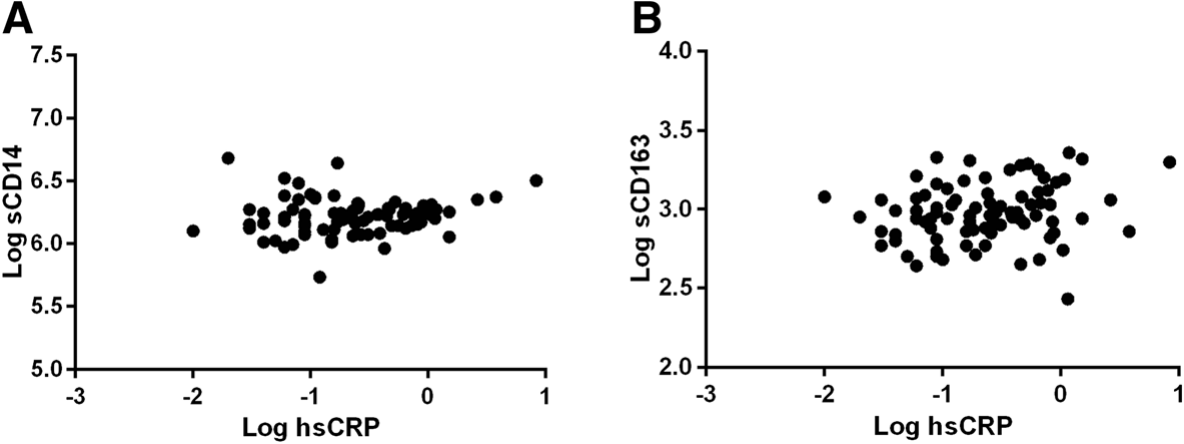
**Correlation between systemic inflammation and monocyte activation markers.** Correlation between levels of Log hsCRP and markers of monocyte activation, (**A)** Log sCD14 (p = 0.030, Spearman correlation) and **(B)** Log sCD163 (p = 0.022) measured in the cohort (n = 84).

We assessed the influence of CD14 C-260T SNP on markers of monocyte activation and systemic inflammation. There was no significant difference in the concentration of sCD14 between CC/CT vs TT genotype carriers (p = 0.266) but TT genotype carriers had significantly higher sCD163 levels compared to CC/CT carriers (p = 0.008) (Table 2). The associations did not change when controlling for the effects of smoking, current CD4 T-cell counts, age and gender, factors previously shown to strongly influence monocyte/macrophage activation levels [38-40]. There was no significant difference in the median concentration of hsCRP in the two groups, however hsCRP was significantly correlated with levels of sCD14 (p = 0.030) and sCD163 (p = 0.022) (Figure 1). These associations remained unchanged when analysis was done using a recessive model (CC vs CT/TT, data not shown).

**Table 2:** **Comparison of markers of immune activation, inflammation and cardiovascular risk factors among the CD14 C-260T CC/CT vs TT carriers**

	**CC/CT (n = 56)** ^*^	**TT (n = 24)** ^*^	**p**	**p** ^**Δ**^
**Immune activation and inflammatory markers**				
Log sCD14	6.20 (6.11–6.28)	6.24 (6.15–6.30)	0.266^§^	0.188
Log sCD163	2.94 (2.84–3.07)	3.03 (2.97 3.20)	0.008^§^	0.013
Log hsCRP	-0.77 (-0.32 - -1.08)	-0.53 (-0.26 - -0.95)	0.296^§^	0.232
**Cardiovascular risk factors**				
Body mass index, kg/m^2^	22.4 (20.9–25.2)	23.9 (20.6–26.0)	0.482^§^	
Fasting sugar, mmol/L	5.0 (4.6–5.5)	5.1 (4.6–5.8)	0.298^§^	
Total cholesterol, mmol/L	5.2 (4.6–6.0)	5.1 (4.8–5.6)	0.858^§^	
Triglyceride, mmol/L	1.8 (1.4–2.3)	2.0 (1.5–2.8)	0.239^§^	
HDL, mmol/L	1.24 (0.99–1.49)	1.12 (0.90–1.31)	0.087^§^	
LDL, mmol/L	2.9 (2.5–3.8)	3.1 (2.6–3.5)	0.978^§^	
Age, years	41 (36–46)	41 (36–48)	0.961^§^	
Male gender, %	82.1%	70.8%	0.257^#^	
Current smoker, %	30.4%	33.3%	0.792^#^	
Framingham risk score	5.05 (2.80–9.08)	4.75 (2.23–14.80)	0.925^§^	

*Data shown are median (IQR) unless otherwise stated; ^§^p-value for Mann-Whitney U; ^#^p-value for Chi-square test; ^Δ^p-value adjusted for smoking status, current CD4 T-cell counts, age and gender.

### Association between CD14 C-260T SNP and increased cIMT

We next assessed if the CD14 C-260T SNP was independently associated with increased cIMT using a multivariate regression model adjusting for both HIV-specific clinical parameters and traditional cardiovascular risk factors. In univariable analysis, we found only increasing age and Framingham score were significantly associated with increased cIMT, while log current CD4 T-cell counts and log sCD163 levels were candidate predictors based on p-value thresholds set *a priori*, p < 0.20. In the multivariable model however, only higher Framingham risk score was independently associated with higher cIMT while there was a trend that lower log sCD163 levels was associated with increased cIMT (Table 3).

**Table 3:** **Risk factors associated with carotid intima media thickness (cIMT) using univariable and multivariable models**

**Predictors**	**Co-efficient (95% confidence interval)**	**p-value**
**Univariable model**		
Age, years	0.007 (0.004, 0.011)	<0.001*
Body mass index, kg/m^2^	0.006 (-0.005, 0.017)	0.273
Total cholesterol, mmol/L	0.015 (-0.021, 0.051)	0.415
Triglyceride, mmol/L	0.003 (-0.029, 0.029)	0.985
HDL, mmol/L	-0.001 (-0.105, 0.103)	0.984
LDL, mmol/L	0.019 (-0.022, 0.060)	0.369
Fasting glucose, mmol/L	-0.017 (-0.053, 0.020)	0.366
Framingham score, %	0.011 (0.006, 0.016)	<0.001
Baseline CD4 T-cell counts, per 100 cells/μl	-0.011 (-0.044, 0.022)	0.527
Log baseline HIV RNA	-0.006 (-0.028, 0.016)	0.585
Log current CD4 T-cell counts	0.043 (-0.016, 0.104)	0.151
Log sCD14	0.020 (-0.083, 0.123)	0.700
Log sCD163	-0.054 (-0.135, 0.027)	0.190
Log hsCRP	0.0005 (-0.025, 0.026)	0.970
CD14 C-260T genotype; CC/CT vs TT	-0.013 (-0.099, 0.071)	0.758
**Multivariable model**		
Framingham score, %	0.009 (0.004, 0.014)	<0.001
Log current CD4 T-cell counts	0.038 (-0.033, 0.108)	0.289
Log sCD163	-0.084 (-0.182, 0.013)	0.087

*Age was omitted from the multivariable model given that it is one of the components used to derive the Framingham risk score.

## Discussion

This is the first study to assess the potential modifying effect of the CD14 C-260T SNP on monocyte activation and inflammation in HIV-infected individuals. In this clinic-based cohort of HIV-infected patients on cART who had a no clinical history of CVD, diabetes, hypertension and dyslipidemia at recruitment, 27% had evidence of sub-clinical atherosclerosis defined by increased cIMT >0.7 mm. The CD14 C-260T T-allele was the major allele carried in 56% of the cohort and consistent with the reported distribution among Asians in Hapmap. In a multivariable analysis, the CD14 C-260T SNP was however not associated with sub-clinical atherosclerosis as measured by cIMT in this treated HIV cohort.

We found that sCD163 was significantly higher in TT homozygous vs CC/CT carriers. Soluble CD163 is a hemoglobin-heptoglobin scavenging receptor that is shed from monocyte/macrophages following pro-inflammatory stimulation including LPS [41,42] and therefore considered a marker of monocyte activation. Functionally however, sCD163 has been described to have a role in attenuating inflammatory processes (reviewed in [43]). In HIV-infected individuals, sCD163 levels positively correlated with monocyte and T-cell activation and inversely with CD163 expression on CD14 + CD16+ monocytes [44]. To date, we have not found any prior studies that have assessed the association between the CD14 C-260T SNP and sCD163 in the general population. Our findings that sCD163 are increased with the CD14 C-260T TT genotype implies that carriers of this genotype have increased monocyte activation.

Prior studies in HIV-infected individuals on cART found increased monocyte activation was an independent predictor of CVD [10,34,35,45-48] and mortality [49]. A recent study found that monocyte activation and not T-cell activation was strongly associated with markers of systemic inflammation and coagulation (IL-6, hsCRP and D-dimers) which has been shown to predict many serious non-AIDS events (SNAEs) and mortality in HIV-infected individuals [50]. Therefore in HIV-infected individuals, TT genotype carriers may be at a higher risk of SNAEs due to increased monocyte activation. Larger prospective studies assessing the association of this SNP with more detailed characterisation of monocyte activation markers and clinical end-points will be needed to confirm this.

In this study, we found no significant difference in sCD14 levels in homozygous TT vs CC/CT carriers with and without adjusting for current CD4 T-cell counts, smoking status, age and gender. The lack of statistical significance could potentially be due to the small sample numbers in our study or due to the influence of other SNPs not measured in this study that could have additional modulating effects on sCD14 levels. A recent genome wide association study (GWAS) in 5000 older individuals (>65 years) identified 164 SNPs which were associated with sCD14 levels and responsible for approximately 33% of the phenotypic variance [13]. Some studies of the CD14 C-260T SNP in the general population have found T vs C allele carriers had increased membrane-bound CD14 and sCD14 [18,19,22] though not all studies have found this association [30,51]. *In vitro* studies have also found the T vs C allele was associated with increased inducible sCD14, TNFα and IL-6 production following LPS-stimulation of whole blood [19,21,23] but we did not measure these parameters in this study.

We did not find a significant difference in the median levels of hsCRP in TT vs CC/CT carriers. Increases in hs-CRP have been shown to correlate with the extent of vascular disease measured by the presence of vascular dysfunction in multiple vascular beds (carotid, coronary and brachial artery) [34]. Indeed, most studies that have found an association between hsCRP and the CD14 C-260T SNP [23,52,53] have so far been in populations that have already developed cardiovascular disease and unlike our study participants who had subclinical disease. This difference in cohort characteristic may have precluded our ability to show significant differences by genotype though hsCRP was significantly correlated with both sCD14 and sCD163 levels.

Only increasing age and Framingham risk score were significantly associated with cIMT in univariable analysis while log current CD4 T-cell counts and log sCD163 levels were candidate predictors. In the multivariable model however only increased Framingham risk score was significantly associated with increased cIMT while log sCD163 and log current CD4 T-cell count were not significantly associated with cIMT levels. Framingham risk score is a composite index of multiple established risk factors which are individually associated with CVD risks and therefore its association with cIMT in HIV-infected individuals is expected as previously found [54]. Prior studies in HIV-infected individuals have found sCD163 levels to be associated with non-calcified plaques [45,47], increased progression of cIMT [10] and coronary artery calcification [35], though not all studies have reported an association with CVD as we have found [55]. The exact role of sCD163 as a driver of atherosclerosis progression is unclear. Though sCD163 is a marker of increased monocyte activation, it has also been described to have an anti-inflammatory role by increasing the production of IL10 and inhibiting T-cell activation [56,57].

The CD14 C-260T SNP was not significantly associated with cIMT in this cohort. Prior studies have reported both significant [17-19,22] and non-significant associations [29,38,58] between the C-206T SNP and cIMT. Our failure to demonstrate a significant association if one truly existed could have been affected by the small sample size (power = 33%) and the low CVD risks in this cohort. Indeed, all prior studies in East Asians that have reported a significant association between the CD14 C-260T SNP and CVD were in populations of high CVD risks [33].

There were a number of important limitations in our study. First, we only genotyped the CD14 C260T SNP in this study while numerous other SNPs have recently been described to additionally modulate sCD14 levels [13]. In contrast to other SNPs associated with CVD, the CD14 C-260T SNP has been associated with multiple other inflammatory diseases [59-62] consistent with involvement in important non-redundant biological pathways. Second, many of our findings were probably confounded by the small sample numbers in our cohort. The recruitment in our study was dependent on patients re-consenting for genetic testing from an established clinic-based cohort of patients reporting no clinical history of CVD risk factors. We only had 33% power to show a significant association of cIMT with this SNP if one existed (at the 5% significance level, presuming a ratio of 1:1) taking into account the average increased attributable risk to the T-allele in non-HIV infected East Asians [33] and the baseline rate of events (defined as increased cIMT >0.7 mm) in C-allele carriers in our cohort.

## Conclusion

This is the first study to assess the relationship between the CD14 C-260T SNP and monocyte activation, inflammation and cIMT in HIV-infected individuals. We found that the CD14 C-260T SNP was associated with increased monocyte activation but not systemic inflammation or cIMT in this HIV-infected cohort with low CVD risk profile. Given that drivers of chronic immune activation persist despite cART and that increased monocyte activation has been associated with morbidity and mortality in HIV-infected patients on cART, the potential influence of this SNP in chronic HIV disease warrants further investigation.

## Abbreviations

HIV: Human immunodeficiency virus
CVD: Cardiovascular disease
SNP: Single nucleotide polymorphism
UMMC: University Malaya Medical Centre
hsCRP: Highly sensitive C-reactive protein
sCD14: Soluble CD14
sCD163: Soluble CD163
TLR4: Toll-like receptor
cIMT: Carotid intima media thickness
cART: Combination antiretroviral therapy
LPS: Lipopolysaccharide
IQR: Inter-quartile range
NNRTI: Non-nucleoside reverse transcriptase inhibitor
NRTI: Nucleoside reverse transcriptase inhibitor
PI: Protease inhibitors
SNAE: Serious non-AIDS events
AIDS: Acquired immunodeficiency syndrome
